# It's All about Timing: The Involvement of Kir4.1 Channel Regulation in Acute Ischemic Stroke Pathology

**DOI:** 10.3389/fncel.2018.00036

**Published:** 2018-02-16

**Authors:** Meagan Milton, Patrice D. Smith

**Affiliations:** Department of Neuroscience, Carleton University, Ottawa, ON, Canada

**Keywords:** Kir4.1, ischemia, astrocytes, autophagy, mammalian target of rapamycin

## Abstract

An acute ischemic stroke is characterized by the presence of a blood clot that limits blood flow to the brain resulting in subsequent neuronal loss. Acute stroke threatens neuronal survival, which relies heavily upon proper function of astrocytes. Neurons are more susceptible to cell death when an astrocyte is unable to carry out its normal functions in supporting the neuron in the area affected by the stroke (Rossi et al., 2007; Takano et al., 2009). For example, under normal conditions, astrocytes initially swell in response to changes in extracellular osmotic pressure and then reduce their regulatory volume in response to volume-activated potassium (K^+^) and chloride channels (Vella et al., 2015). This astroglial swelling may be overwhelmed, under ischemic conditions, due to the increased levels of glutamate and extracellular K^+^ (Lai et al., 2014; Vella et al., 2015). The increase in extracellular K^+^ contributes to neuronal damage and loss through the initiation of harmful secondary cascades (Nwaobi et al., 2016). Reducing the amount of extracellular K^+^ could, in theory, limit or prevent neuronal damage and loss resulting in an improved prognosis for individuals following ischemic stroke. Kir4.1, an inwardly rectifying K^+^ channel, has demonstrated an ability to regulate the rapid reuptake of this ion to return the cell to basal levels allowing it to fire again in rapid transmission (Sibille et al., 2015). Despite growing interest in this area, the underlying mechanism suggesting that neuroprotection could occur through modification of the Kir4.1 channel's activity has yet to be described. The purpose of this review is to examine the current literature and propose potential underlying mechanisms involving Kir4.1, specially the mammalian target of rapamycin (mTOR) and/or autophagic pathways, in the pathogenesis of ischemic stroke. The hope is that this review will instigate further investigation of Kir4.1 as a modulator of stroke pathology.

## General properties of Kir4.1

Kir4.1, initially named BIR10, was first identified by the Adelman's group (Bond et al., 1994). It is predominately expressed on glial cells and is responsible for developmental regulation of extracellular K^+^ dynamics (described below) (D'Adamo et al., 2011, 2013). Research has demonstrated that the channel forms both homomeric Kir4.1-Kir4.1 tetramers and heteromeric Kir4.1-Kir5.1 tetramers (Hibino et al., 2010; D'Adamo et al., 2011, 2013). The two types of tetramers have different sensitivities to pH. Between 6.5 and 8.0, the homomeric Kir4.1 channel is inhibited, whereas the heteromeric Kir4.1-Kir5.1 is suppressed significantly (Pessia et al., 2001; D'Adamo et al., 2011, 2013). In addition, Kir4.1 and Kir5.1 are coexpressed on locus coeruleus neurons where they appear to be involved in neuronal carbon dioxide (CO_2_) chemosensitivity (D'Adamo et al., 2011). Taken together, these results suggest that pH, CO_2_, and Kir5.1 subunits modulate Kir4.1 activity.

Previous research has demonstrated that Kir4.1 channels are implicated in the pathophysiology of several disease/disorders. Abnormalities, specifically missense variations, within this channel have been linked to epilepsy (Hibino et al., 2010; D'Adamo et al., 2013). For example, a missense variation in Kir4.1 (T262S) was found to be the reason that DBA/2 mice are more susceptible to induced seizures than C57BL/6 mice (Ferraro et al., 2004; D'Adamo et al., 2013). In patients with either focal or generalized epilepsy, the R271C mutation in the Kir4.1 channel has been associated with a resistance to seizures (Ferraro et al., 2004; D'Adamo et al., 2013). Another condition, autism spectrum disorders (ASD), is associated with Kir4.1 channel mutations. In children with ASD, two specific mutations (R18Q and V84M) within the Kir4.1 channel have been identified. R18Q has been associated with several ASD symptoms, such as absence of speech and severe social interaction deficits, whereas the V84M appears to confer an increased expression of poor social gaze and withdrawal behaviors that are also characteristic of ASD (Sicca et al., 2011; Guglielmi et al., 2015). Kir4.1 abnormalities are also believed to underlie the comorbidity between epilepsy and ASD (D'Adamo et al., 2013; Guglielmi et al., 2015). Finally, Kir4.1 activity appears to be altered in response to ischemia (Nwaobi et al., 2016). Research has shown that Kir4.1 expression and Kir-mediated currents are reduced from day 1 to day 14 post-injury following global and focal ischemia (Pivonkova et al., 2010; Steiner et al., 2012). Furthermore, Kir4.1 channels appear more at the soma of the astrocyte rather than on the astrocytic processes when these reductions occur (Stewart et al., 2010; Nwaobi et al., 2016). It has been suggested that this shift changes Kir4.1 focus to proliferation instead of K^+^ spatial buffering (Nwaobi et al., 2016).

## The role of Kir4.1 in astrocytic functioning

Within the central nervous system, astrocytes are involved in controlling ion and water homeostasis, moving metabolite and waste products, and participating in the formation of the blood-brain barrier (Takano et al., 2009; Nwaobi et al., 2016). These biophysical properties appear to be affected by Kir4.1; specifically, the homeostasis of extracellular K^+^, the regulation of extracellular glutamate, and the mediation of water and volume levels are three of the processes that Kir4.1 is involved (Nwaobi et al., 2016). First, K^+^ spatial buffering is a process carried out by astrocytes to ensure that extracellular K^+^ concentrations are regulated following an action potential (Nwaobi et al., 2016). An action potential may increase local K^+^ concentrations by 1 mM under normal neuronal activity or by >10–12 mM under ischemic conditions (Moody et al., 1974; Ransom et al., 2000). The increased K^+^ concentrations prevent the neuron from firing in response to further stimulation and in the case of ischemic conditions, contribute to neuronal loss (Nwaobi et al., 2016). Thus, it is important for K^+^ to be removed from the extracellular space through a mechanism such as the Kir4.1 channel. This is supported by the fact that at the sites of local accumulation, Kir4.1 has been shown to allow K^+^ influx to return the extracellular K^+^ concentrations to baseline. Recent work has demonstrated that local extracellular K^+^ concentrations were greater following the blockage of Kir4.1 using barium (Ransom et al., 2000; Larsen et al., 2014; Nwaobi et al., 2016). Furthermore, previous work has shown a slower recovery rate of extracellular K^+^ concentrations and enhanced undershoot recovery following brainstem stimulation in glial-conditional Kir4.1 knock out animals (Neusch et al., 2006). Taken together, these results implicate the Kir4.1 channel's involvement in extracellular K^+^ homeostasis and neuronal survival following ischemia.

Second, astrocytes are involved in glutamate uptake through two transporters, GLAST (EAAT1) and GLT-1 (EAAT2), in an energetically unfavorable process (Rothstein et al., 1996). Sodium, hydrogen and K^+^ electrochemical gradients work in concert with the two transporters to bring glutamate into the astrocyte in an efficient manner (Barbour et al., 1988; Nwaobi et al., 2016). With respect to extracellular K^+^, high concentrations decrease K^+^ unbinding and depolarize the glial membrane reducing the amount of glutamate reuptake resulting in an increase in neuronal excitability (Barbour et al., 1988). The contribution of Kir4.1 to astrocytic glutamate uptake has been previously studied. Researchers found a 33.1 and 57.0 percent decrease in glutamate uptake following pharmacological inhibition and siRNA-mediated Kir4.1 knockdown in cortical astrocytes (Kucheryavykh et al., 2007). The authors attributed these decreases to be the result of a loss in the hyperpolarized resting membrane potential of the astrocyte. In addition, a TBOA (*threo*-beta-benzyloxyaspartate)-sensitive glutamate uptake reduction of >50 percent was seen in Kir4.1 knockout animals when compared to wild-type animals (Nwaobi et al., 2016). The results support Kir4.1's role in glutamate uptake by allowing the astrocyte to maintain a K^+^ electrochemical gradient that promotes K^+^ unbinding and glial depolarization.

Lastly, a third property of astrocytes that appears to involve Kir4.1 functioning is water and volume regulation. Aquaporins, specifically aquaporin 4 (AQP4), are found at the astroglial endfeet where they are responsible for bringing water into specific cells and removing excess water to alleviate swelling to prevent cell lysis (Vella et al., 2015). Under conditions of water and/or food deprivation, AQP4 has demonstrated an ability to alter its expression levels in order to maintain the brain's normal water content and prevent cell loss (Ye et al., 2016). Studies examining the relationship between Kir4.1 and AQP4 have found that they co-localize with one another. For example, one study demonstrated that co-immunoprecipitates between the two were found in Müller cells and that loss or mislocalization of Kir4.1 in the post-ischemic retina played a role in glial cell swelling (Pannicke et al., 2004). However, a follow-up study was able to induce the same cell swelling through the inhibition, using physostigmine, of Kir4.1 to prevent K^+^ efflux without changing AQP4 expression or function (Nwaobi et al., 2016). This suggests that changing Kir4.1 channel's activity alone, through the application of different pharmacological agents, could reduce the amount of cell lysis or autophagic cell death seen in ischemic conditions.

**Figure 1:**
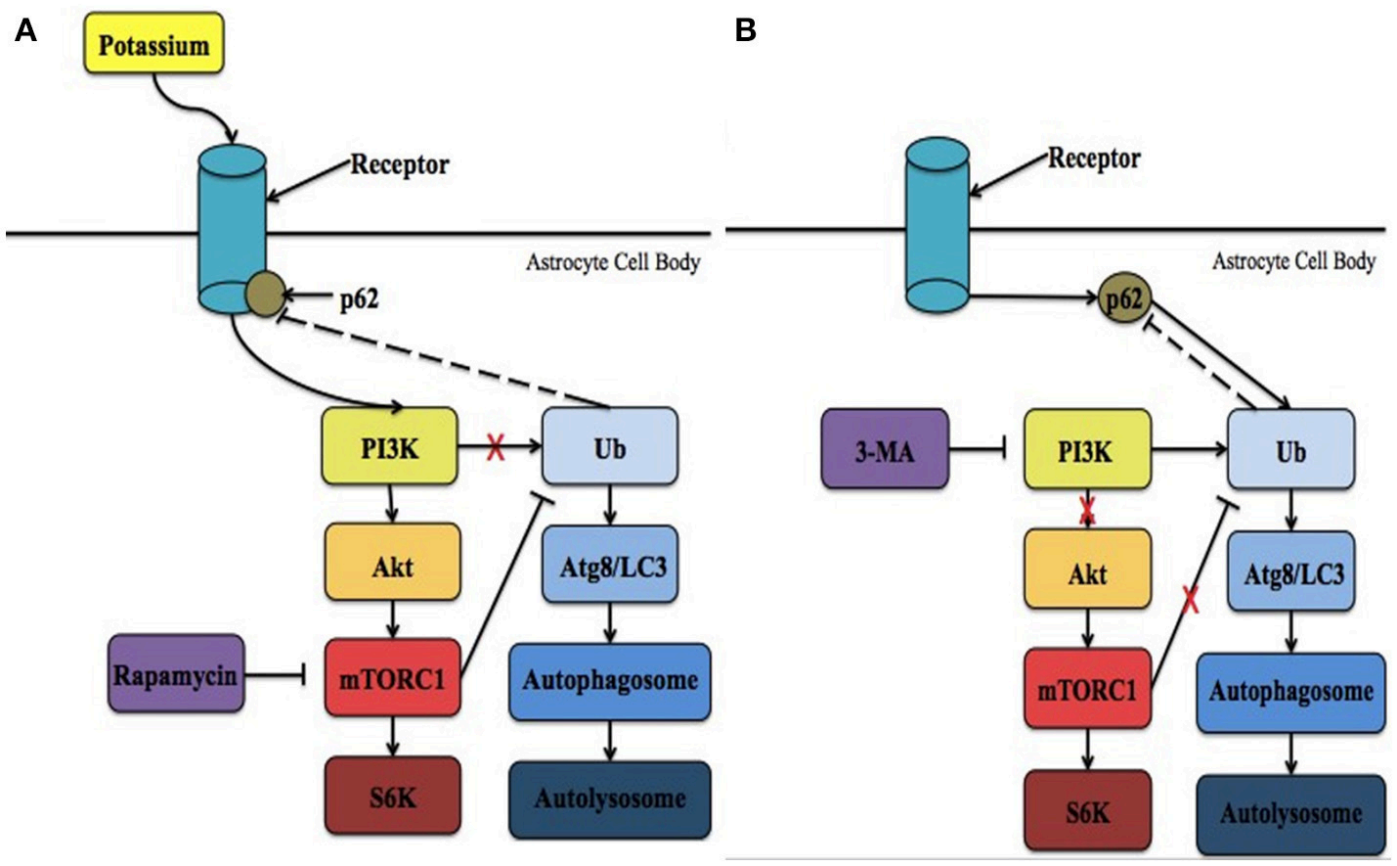
The proposed intrinsic mechanism involving Kir4.1 in neuronal survival following ischemia. **(A)** Following ischemia, the levels of extracellular potassium increase significantly. PI3K is recruited to activate Kir4.1 leading to the initiation of mTORC1 and the overall mTOR pathway. In addition, it is possible that p62 is recruited to regulate Kir4.1. As a result, more cells in the ischemic penumbra survive and brain injury is reduced. Treatment with rapamycin has been demonstrated to reduce these effects. **(B)** During potassium starvation Kir4.1 is not activated due to ATP-depletion and mTORC1 no longer negatively regulates autophagy. Instead, autophagic cell death via protein breakdown in the autolysosome is initiated in a PI3K-dependent manner. Under these conditions, p62 interacts with ubiquitin (Ub) and binds with Atg8/LC3 within the phagophore. Lysosomes then interact with the autophagosome to form the autolysosome. 3-MA is an inhibitor of the PI3K pathway that prevents the initiation of autophagic cell death. Ubiquitin is the only molecule that is depicted as potentially feeding back in the mechanism, however, many other molecules are known to feedback in both the mTOR and autophagic pathways, respectively.

In addition, due to the interaction between Kir4.1 and APQ4 channels, an important factor that may modulate Kir4.1 activity is water movement through different compartments. With a specific focus on glutamate, a review published by Rothman and Olney (1986) described how neurotoxicity results in the simultaneous build-up of intracellular sodium, K^+^, and water that may lead to lysis/autophagic injury. Glutamate, a neurotransmitter impacted by K^+^ gradients and Kir4.1 as described above, acts on many different receptors to exert its effects. The two most important ones for the purpose of this review are α-amino-3-hydroxy-5-methyl-4-isoxazolepropionic acid (AMPA) and kainite (KA) receptors. These two receptors are highly permeable to sodium and K^+^ (Chen et al., 1998). Over-activation of these receptors, as well as the *N*-methyl-D-aspartate (NMDA) receptor, results in response to brain ischemia (Rothman and Olney, 1987; Coyle and Puttfarcken, 1993). The over-activation leads to excessive ion influx, osmotic swelling, free radical generation, and cell death (Rothman and Olney, 1987; Coyle and Puttfarcken, 1993). Specifically, Rothman and Olney (1986) found that the recovery of water regulation might also reverse the lysis/autophagic injury. Based on these findings, water accumulation may also be considered a “stressor” that damages organelles. Due to the Kir4.1 channel's interaction with APQ4, and its involvement in water/volume regulation, further studies are required to examine how levels of intracellular K^+^ contribute to such conditions and how the proposed mTOR pathway involving Kir4.1 (described below; Figure 1) is linked to cell survival.

## Potential mechanisms involved in mediating Kir4.1 functional benefits

Kir4.1 channel activity following ischemia and its contribution to the biophysical properties of astrocytes has previously been described. How Kir4.1 is involved in preventing neuronal loss following ischemia through an intrinsic pathway has yet to be outlined. Specifically, it is possible that Kir4.1 may play a role in the activation of the mammalian target of rapamycin complex 1 (mTORC1). mTORC1 is one of two mTOR complexes that contains a kinase mTOR component (Heras-Sandoval et al., 2014). It is mediated upstream by the phosphatidylinositol 3 kinase/protein kinase B (PI3K/AKT) pathway that is involved in cell survival and the inhibition of apoptosis (Heras-Sandoval et al., 2014). Furthermore, researchers have shown that mTORC1 is involved in the negative regulation of autophagy (Noda and Ohsumi, 1998; Schmelzle and Hall, 2000; Cuyàs et al., 2014).

Autophagy involves the breakdown of damaged organelles and misfolded proteins *via* a stress-induced catabolic pathway that maintains proper cellular homeostasis (Heras-Sandoval et al., 2014; Sahni et al., 2017). Autophagy does not seem to always result in cell survival. Autophagic programmed cell death occurs in response to stressors, such as water accumulation or nutrient deprivation, due to induced autophagy (Heras-Sandoval et al., 2014). Induced autophagy has been shown to occur in response to altered expression of autophage-related gene (Atg) 5 and 6 within the cell leading to cellular lysis (Amelio et al., 2011; Majid, 2014). Research has also demonstrated that autophagy is impacted significantly more in nutrient-deprived situations, such as K^+^-deprivation, as the process is associated with energy re-usage in cells (Ye et al., 2016; Sahni et al., 2017). For example, within cerebellar granule cells, K^+^-deprivation has not only induced autophagy but has been linked to programmed cell death as conditions move into K^+^-starvation (K^+^ reduced to 5 mM) (Canu et al., 2005; Kaasik et al., 2005; Sahni et al., 2017). Kir4.1 is dependent on adenosine triphosphate (ATP) (Nwaobi et al., 2016). Under K^+^-starvation, Kir4.1 may be inactive as a result of ATP depletion in response to brain ischemia and low pH due to the acidosis that occurs in response to ischemia (Pessia et al., 2001; Hu and Song, 2017). As a result, Kir4.1 is no longer activated in a PI3K-dependent manner (as suggested below) and mTORC1 no longer prevents autophagic cell death.

The point at which PI3K attempts to activate Kir4.1 appears to be dependent on timing. This may be because recent evidence has pointed not only to the dual role of autophagy following ischemia (Chen et al., 2014; Majid, 2014) but implicates the potential role of K^+^ in preventing autophagy (Canu et al., 2005; Kaasik et al., 2005; Sahni et al., 2017). Initially, Koike et al. (2008) demonstrated that the induction of autophagy, following hypoxia-ischemia injury, results in neuronal death. On the other hand, Carloni et al. (2010) described a pro-survival signaling complex involving autophagy to prevent neuronal death. More recently, it was suggested that the role autophagy plays following ischemia is determined by the time at which it is induced (Chen et al., 2014). Ravikumar et al. (2010) stated that a protective role for autophagy might be seen during ischemic preconditioning, whereas following ischemia/reperfusion the process might aggravate cerebral ischemic injury. Based on these findings, He et al. (2012) hypothesized that inducing autophagy at different time points during early and late stage ischemia may account for the different outcomes. For example, infarct size was reduced significantly and eliminated water content increases in the brain after treatment with 3-MA (a known autophagy inhibitor) prior to reperfusion (Chen et al., 2014). On the other hand, Carloni et al. (2010) found that treatment with rapamycin decreased brain injury and increased autophagy when administered prior to hypoxia-ischemia. Furthermore, the neuroprotective effects of ischemic postconditioning, previously described as being mimicked (Yan et al., 2011), are weakened when rapamycin is applied at the onset of reperfusion rather than at the onset of hypoxia-ischemia (Gao et al., 2012).

The mammalian target of rapamycin (mTOR) pathways is one of several cellular pathways that are involved in the maintenance of neuronal survival. It is also inhibited by rapamycin. As mentioned above, the timing at which PI3K attempts to activate Kir4.1, resulting in mTORC1 activation, may determine which pathway is activated leading to either cell survival or death. It is possible that targeting Kir4.1 activity prior to reperfusion may increase PI3K activity, and subsequent AKT phosphorylation, resulting in the activation of the mTORC1. It is important to note that the channel's involvement in the mTOR pathway has only been previously implicated by a single study. Zaika et al. (2016) showed that using insulin and insulin-like growth factor-1, within the cortical collecting duct, activates the heteromeric Kir4.1/Kir5.1 channel in a phosphoinositide 3-kinase (PI3K)-dependent manner. It is possible that Kir4.1 works through the mTOR pathway to prevent the induction of autophagy, through its PI3K-dependent activation (Zaika et al., 2016), following the onset reperfusion leading to the reduction of infarct size and a decrease in water content. This hypothesis is supported by the fact that during the onset of reperfusion ATP levels are high (Murphy and Steenbergen, 2008; Kalogeris et al., 2012), which corresponds to the time when Kir4.1 is optimally activated (Takumi et al., 1995; Kucheryavykh et al., 2009) and APQ4 levels remain unaltered (Shin et al., 2011). On the other hand, when energy levels are low, corresponding to Kir4.1 inactivation, the PI3K/AKT pathway is less active leading to the adoption of a quiescent state by the cells (Cheung and Rando, 2013). Thus, it is hypothesized that Kir4.1 is activated in PI3K-dependent manner, leading to cell survival through the mTOR pathway. Future research could focus on determining the upstream effectors within this intrinsic pathway and suggests that Kir4.1 represents a potential therapeutic target for the treatment of ischemic insult. The two potential mechanisms involved in mediating Kir4.1 functional benefits are summarized in Figure 1.

It is also important to note that p62, an adapter molecule in autophagy, may also play a role in this pathway. Voltage-gated K^+^ channels (K_v_) are regulated by p62 (Sahni et al., 2017). It has been previously demonstrated that PKCζ interacts with p62 to increase the phosphorylation of the β subunit of K_v_ (K_v_β) (Puls et al., 1997; Ishii et al., 2013). As a result, K_v_ channels, specifically K_v_1.5, are inhibited under acute hypoxia (Ishii et al., 2013). While Kir4.1 is not a voltage-gated channel, as it lacks a voltage-sensing domain, PKC isoforms have been shown to modulate both inwardly rectifying and voltage-gated channels (Dini et al., 2006). For example, the heteromeric Kir4.1-Kir5.1 channel is inhibited in a PKC-dependent manner (Rojas et al., 2007). Assuming p62 and Kir4.1 do interact, the timing at which p62 attempts to interact with Kir4.1 may determine which pathway is activated leading to either cell survival or death. Immediately following ischemic onset, p62 may be recruited to regulate Kir4.1, which is activated in a PI3K-dependent manner, leading to cell survival through the mTOR pathway (Figure 1A). Thus, it is possible that targeting Kir4.1 prior to reperfusion, with different pharmacological agents, may increase the likelihood of p62 associating with Kir4.1 and subsequent cell survival. On the other hand, the point at which p62 attempts to interact with Kir4.1 would be dependent on timing. This is because as more ATP is used, Kir4.1 is no longer active and p62 would be able to associate with ubiquitin to initiate autophagic cell death under nutrient-deprived situations (Figure 1B). Future research should examine whether or not the homomeric Kir4.1 channel is inhibited by PKC isoforms, specifically PKCζ, and if there is an interaction between Kir4.1 and p62.

## Conclusion

Previous evidence points to an intrinsic pathway, involving the regulation of Kir4.1, within the central nervous system that regulates neuronal survival. Further understanding of this pathway within astrocytes, and how it impacts neuronal viability (example: the impact on autophagy), could improve treatment options following ischemia. In this regard, further research is critical to identifying the potential role of Kir4.1 channel, and its subsequent effectors, in ischemia in order to guide the development of novel treatment options for stroke. These treatment strategies could be focused on altering Kir4.1 channel activity, which may be useful in improving the clinical outcomes after ischemic stroke.

## Author contributions

All authors listed have made a substantial, direct, and intellectual contribution to the work, and approved it for publication.

### Conflict of interest statement

The authors declare that the research was conducted in the absence of any commercial or financial relationships that could be construed as a potential conflict of interest.

## Abbreviations

AKT: protein kinase B
AMPA: α-amino-3-hydroxy-5-methyl-4-isoxazolepropionic acid
ASD: autism spectrum disorders
ATG: autophagy-related gene
ATP: adenosine triphosphate
AQP4: aquaporin 4
CO_2_: carbon dioxide
GLAST: L-glutamate/L-aspartate transporter
GLT-1: glutamate transporter 1
K^+^: potassium
KA: kainite receptor
mTOR: mammalian target of rapamycin
mTORC1: mammalian target of rapamycin complex 1
NMDA: *N*-methyl-D-aspartate
PI3K: phosphatidylinositol 3 kinase.
